# Ferromagnetic Semiconductor In_0.75_Fe_0.25_Sb for All-Optical Control of Light Polarization

**DOI:** 10.1021/acsomega.5c10405

**Published:** 2026-03-16

**Authors:** Matheus Franco Ribeiro, Juliana Zarpellon, Dante Homero Mosca, José Varalda

**Affiliations:** † Departamento de Física, 28122 Universidade Federal do Paraná, Caixa Postal 19044, Curitiba CEP 81531-990, Paraná, Brazil; ‡ Centro Interdisciplinar de Ciência, Tecnologia e Inovação, Curitiba CEP 81530-001, Paraná, Brazil; ¶ Programa de Pós-Graduação em Engenharia e Ciência dos Materiais, Universidade Federal do Paraná, Caixa Postal 19011, Curitiba CEP 81531-980, Paraná, Brazil

## Abstract

In this work, we investigate highly iron-doped indium antimonide, whose electronic structure is favorable for use in ultrafast magneto-optical devices. Density functional theory (DFT) calculations show that the incorporation of 25% Fe into InSb gives rise to a degenerate n-type ferromagnetic semiconductor. A high concentration of spin-polarized electrons around the Fermi level and spin splitting of the Fe 3d orbitals with asymmetric occupation of the orbital states due to hybridization with Sb 5p orbitals create a conduction band. Hybridization, with a significant contribution of magnetic moment in interstitial regions, stabilizes the ferromagnetism in the zinc-blende structure. The peculiar bulk inversion asymmetry (BIA) of this structure determines the existence of an intrinsic magnetocrystalline anisotropy with the easy magnetization axis along the crystallographic [111] axis, the only preserved inversion symmetry axis. The intraband transitions between spin-split Fe 3d orbitals generate magnetic circular dichroism and the Kerr effect with rotation reversals and ellipticity in the visible region (1.8 and 2.5 eV) that enable applications in ultrafast devices based on the spin-transfer torque phenomenon and all-optical tunable magnetic Kerr gates based on Kerr dispersion.

## Graphical abstract

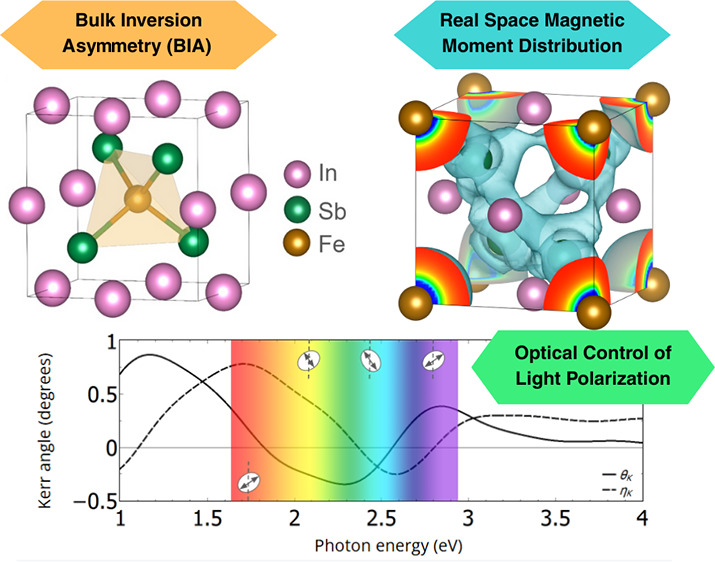

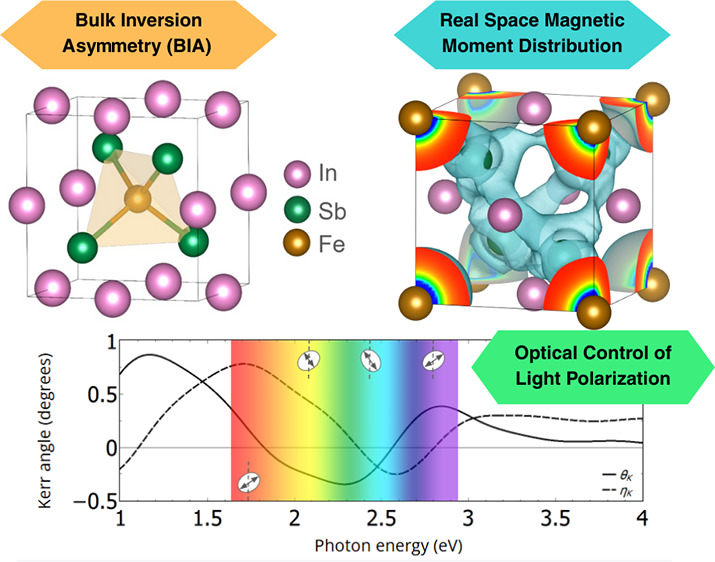

## 1 Introduction

Ferromagnetic semiconductors (FMS) combining ferromagnetism and semiconductor properties are essential for the development of semiconductor-based spintronics.^1–3^ They pave the way for a new generation of spintronic devices based on magnetic control of charge carriers and gate voltage at room temperature such as high-speed transistors and nonvolatile magnetic memories that are energy-saving and compatible with current semiconductor technology.^4,5^

Seminal work on the great potential for applications of dilute magnetic semiconductors (DMS) has triggered numerous theoretical and experimental studies.^6–14^ However, theoretical studies based on DFT calculations of the electronic structure of most of the semiconductor or insulating materials doped with transition metals have demonstrated that they do not exhibit intrinsic carrier-mediated ferromagnetism^15,16^ and even the most promising DMS have Curie temperatures below 200 K.^17^ Furthermore, DMS require an intermediate layer at the interface with metals to enable spin transfer channels due to their mismatch in electrical conductivities.^18–20^ Only recently these difficulties and disadvantages have been overcome with the successful preparation of heavily iron-doped semiconductors exhibiting zinc-blende crystal structure. FMS in which iron is used as a magnetic dopant include p-type (Ga,Fe)­Sb,^21–23^ n-type (In,Fe)­Sb,^23–27^ n-type (In,Fe)­As^28–30^ and n-type (Ga,Fe)­As.^31,32^

Despite the very high Fe doping level, these materials prepared as nanometer-thick epilayers are single-phase FMS with the intrinsic mechanism of ferromagnetic ordering without visible evidence of secondary phases.^33,34^ The higher Curie temperatures (*T*_C_) are reported to Ga_0.80_Fe_0.20_Sb with *T*_C_ of about 400 K^35^ and In_1–*x*_,Fe_*x*_Sb with *T*_C_ = 385 K for *x* = 0.35 and *T*_C_ = 335 K for *x* = 0.16.^26^ These FMS are promising for the realization of n-p diode junctions and implementation of bipolar spintronics using epitaxial heterostructures with multicomponent Fe-doped FMS^36,37^ operating at room temperature. However, it should be noted that the character of electronic conduction in FMS can be ambiguous with respect to the signals extracted from ordinary and anomalous Hall effect measurements.^4,25^ Additional magnetic properties are required for FMS to favor the development of semiconductor-based spintronic devices. For spin diodes^38^ and spin transistors,^27,39,40^ magnetic anisotropy is necessary to maintain stable magnetized orientations as well as to control magnetization switching. The spintronic devices based on the anomalous Hall effect also require the spin–orbit coupling present in the valence band of the semiconductor as well as the spin splitting of the energy in the conduction band to induce an efficient charge-spin interconversion.^41^ On the other hand, FMS that exhibit strong magnetic circular dichroism and high Kerr angle rotations under optical excitations are required for the development of opto-spintronic devices.^42–44^ The integration of this FMS with InSb-type interfaces in InSb nanostructures that are already present in devices such as field-effect transistors,^45,46^ single^47,48^ and double quantum dot devices^49,50^ opens a promising path for new optoelectronic devices based on the stabilization and switching of magnetization orientation in planar junction structures, as well as in the acquisition and analysis of infrared (IR) radiation through magnetic circular dichroism and the Kerr rotation angle. Explicitly, this work advances beyond prior DFT studies on (In,Fe)Sb by focusing on the effects of BIA-driven anisotropy on the material’s electronic and magnetic properties, which allows the proposal of a fully optically controlled magneto-optical Kerr gate device.

## 2 Results and Discussion

In order to investigate the structural influence of Fe doping in the InSb lattice, we consider the supercell In_3_FeSb_4_. Although there is experimental evidence for the coexistence of microphases with Fe in real samples,^23,25,27^ our model reproduces an homogeneous In_3_FeSb_4_ bulk where neither finite-size nor overdoping effects are present. DFT calculations are employed to elucidate the ferromagnetism with intrinsic magnetic anisotropy and magneto-optical responses of In_0.75_Fe_0.25_Sb in the range of hundreds of terahertz from Fe 3d intraband transitions. Magnetic circular dichroism (MCD) results are compared with experimental data of similar samples available in the literature.^26,51^

The calculations were made using the all-electron full potential linearized augmented plane wave (FP-LAPW) method as implemented in the ELK code,^52^ considering the supercell In_3_FeSb_4_. The starting conventional unit cell of InSb (space group 216) was constructed by using the experimental lattice parameter *a* = 6.479 Å (ICSD #41445). The GGA exchange–correlation functional within the PBEsol^53^ approximation was used in noncollinear (spin–orbit coupling included) spin-polarized calculations. Pure PBEsol calculations were chosen over hybrid functionals such as HSE06 due to the much lower computational cost of the former while still yielding good predictions of band gap for InSb and lattice parameters for heavily Fe-doped InSb in comparison to published experimental data. The modified tetrahedron method^54^ with a grid of 20 × 20 × 20 *k*-points in the Brillouin zone was used for the integration in reciprocal space. The total energy and the Kohn–Sham potential convergences were better than 1 × 10^–6^ and 1 × 10^–8^ Ha, respectively. Some optical properties and band structure plots were extracted from the ground state using the elk-optics utility.^52^ For optical broadening parameters and thermalization settings a smearing of 0.01 eV was considered.

We started by finding the minimum energy volume (MEV) for InSb and used this as the initial volume to find the MEV for the doped cases. The total cell volumes were varied isometrically and the MEV was evaluated by fitting equations of state (EOS) to energy-volume data.^55^ The resulting MEVs used here for the optical and magneto-optical calculations have the following lattice parameters: InSb (6.528 Å), In_0.75_Fe_0.25_Sb (6.454 Å) and InSb_0.75_Fe_0.25_ (6.472 Å). The energy minimization results show that In_0.75_Fe_0.25_Sb is much more favorable than InSb_0.75_Fe_0.25_, which is why the latter is not considered in this work.

Although the amount of Fe is much greater than the estimated maximum solubility for Fe in bulk, which appears to be of the order of 10^17^ cm^3^ in InSb (in 1 cm^3^, there are 2.94 × 10^22^ atoms),^56^ DFT calculations indicate that In_0.75_Fe_0.25_As preserving the zinc-blende crystal structure is a degenerate n-type semiconductor. In this case the Fermi energy level (*E*_F_) is shifted toward a conduction band, with a relatively large number of electrons in the conduction band. This result is in agreement with microstructures formed by spinodal decomposition reported for several high Curie temperature FMS containing large amounts of Fe atoms as dopants, synthesized by molecular beam epitaxy.^28,57^ Indeed, the calculated spin-resolved density of states (DOS) for pure InSb is consistent with the presence of a very small energy gap (∼0.16 eV) with respect to which the *E*_F_ is shifted 0.56 eV in energy due to Fe incorporation. In the conduction band with minority spin states, Fe 3d orbitals predominate, as shown in Figure 1a. Such a p-d coupling–induced band crossing and the resulting charge transfer from unoccupied Fe 3d to host is crucial to stabilize the ferromagnetism above room temperature, as suggested by Pillai et al.^27^ using a schematic diagram based on isovalent configuration of Fe^3+^ and Sb^3+^ in In_1–*x*_Fe_*x*_Sb samples with *x* = 0.15.

**Figure 1:**
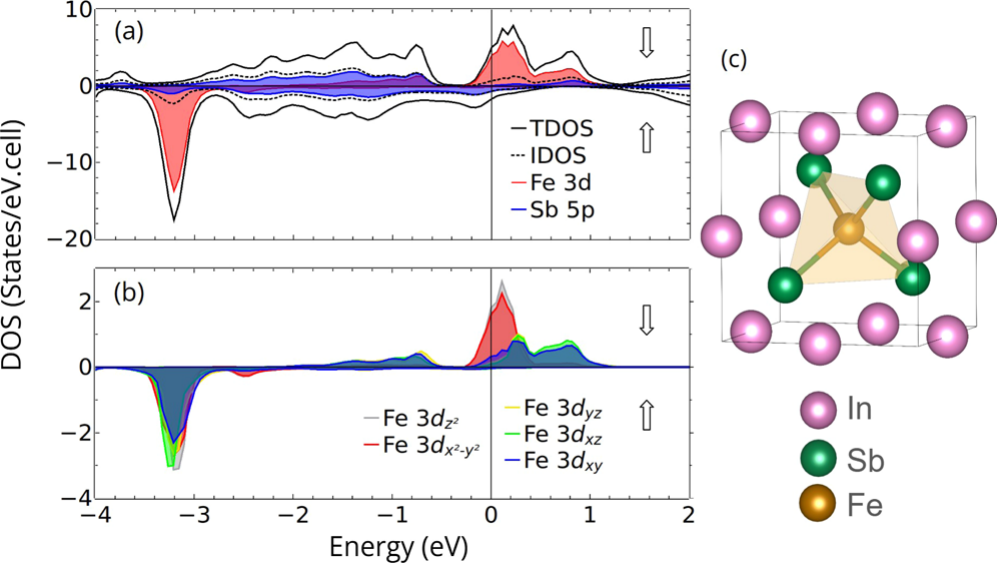
(a) Total density of states (TDOS) for In_0.75_Fe_0.25_Sb, interstitial density of states (IDOS) and local density of states (LDOS) of Fe 3d and Sb 5p, highlighting the spin polarization; (b) partial density of states (PDOS) of Fe 3d in each orbital component. The Fermi level *E*_F_ is set to be 0 eV. (c) The zinc-blende structure of the (In_3_Fe)­Sb_4_ supercell is shown on the right side of the panel, highlighting the iron tetrahedral coordination.

According to the calculations for (In_3_Fe)­Sb_4_ supercell, the Fe atoms are tetrahedrally coordinated by Sb atoms and have 3d orbitals split due to crystal field into components. Dominant at *E*_F_ are Fe 3d_*z* ^2^_ and Fe 3d_*x* ^2^–*y* ^2^_ (*t*_2_ orbitals) with broader Fe 3d_*xy*_, 3d_*xz*_ and 3d_*yz*_ (*e*_g_ orbitals) bandwidths in the valence and conduction bands, as depicted in Figure 1b. Whereas spin-polarized valence band maximum is derived from the Sb 5p orbitals, the p-d coupling–induced band crossing with charge transfer to interstitial region renders a metallic character to In_0.75_Fe_0.25_Sb with a ferromagnetic ground state closely related to the valence band energies of the InSb host. The competition between crystal-field and exchange splittings determines the stability of the high-spin state as the ground state for In_0.75_Fe_0.25_Sb mainly due to the localization of Fe 3d orbitals. Similar results are expected and have already been reported for isovalent dopings of In_1–*x*_Fe_*x*_Sb with *x* around 0.25.^57^

As can be seen in Table 1, the local magnetic moments of the Fe atoms calculated within the muffin-tin spheres is 3.33 μ_B_ and the calculated valence of Fe is +2.2. Clearly, these values are not consistent with those expected for ionic Fe^3+^ (about 5.92 μ_B_) or Fe^2+^(about 4.90 μ_B_). This result corroborates the existence of a complex bonding behavior beyond simple valence states also indicated by the experimental value of 2.76 μ_B_ (with 0.16 μB and 2.60 μ_B_ orbital and spin magnetic moments, respectively) reported for In_0.94_Fe_0.06_Sb using X-ray magnetic circular dichroism measurements.^34^

**Table 1:** Magnetic Moment Per Species in the Unit Cell, As Well As the Spin-Polarized Interstitial Contribution

atomic species	magnetic moment (μ_B_)
In	0.03
Sb	0.03
Fe	3.33
interstitial	0.49
total	4.05

Our results indicate that neither the Zener p-d exchange model,^6,58^ in which itinerant carriers from the conduction band mediate the ferromagnetic p-d exchange interaction, nor an impurity band model can be validated, with localized carriers responsible for hopping conduction mediating the ferromagnetic double exchange interaction,.^59,60^ Indeed, the origin of high *T*_C_ in (In,Fe)­Sb is not due to the carrier induced mechanism because Fe^3+^ does not introduce carriers.^61^ Thus, it is only partially satisfactory to assign the origin of the ferromagnetism and the high *T*_C_ to the double exchange interaction predicted by the impurity band model.^59,60,62^

Real space electronic charge density (ECD) and magnetic moment distribution (MMD) plots for representative isosurfaces are shown in Figure 2. Essentially, BIA determines the formation of interstitial charge dumbbells rotated by 90° with respect to each other when viewed along the [001] and [001̅] directions respectively. Most of the magnetic moment is around Fe atom sites with an intrinsically anisotropic MMD that induces a magnetocrystalline anisotropy. The ECD plot of the (In_3_Fe)­Sb_4_ supercell shown in Figure 2a indicates that the Fe atoms interact only along the diagonals of the faces of the unit cell. This in turn requires the mediation of Sb and In atoms, indicating the crucial role of hybridization. Essentially, Fe atoms are not equivalently connected by Sb along [110] and [11̅0] directions, implying that they are not equivalent with respect to the (001) planes. Therefore, the hybridization of Fe 3d and Sb 5p orbitals generate directional spin polarized charge imbalance in the vicinity of Fe sites. Projective plots of the ECD structure are shown in Figure 2b,c for better visualization.

**Figure 2:**
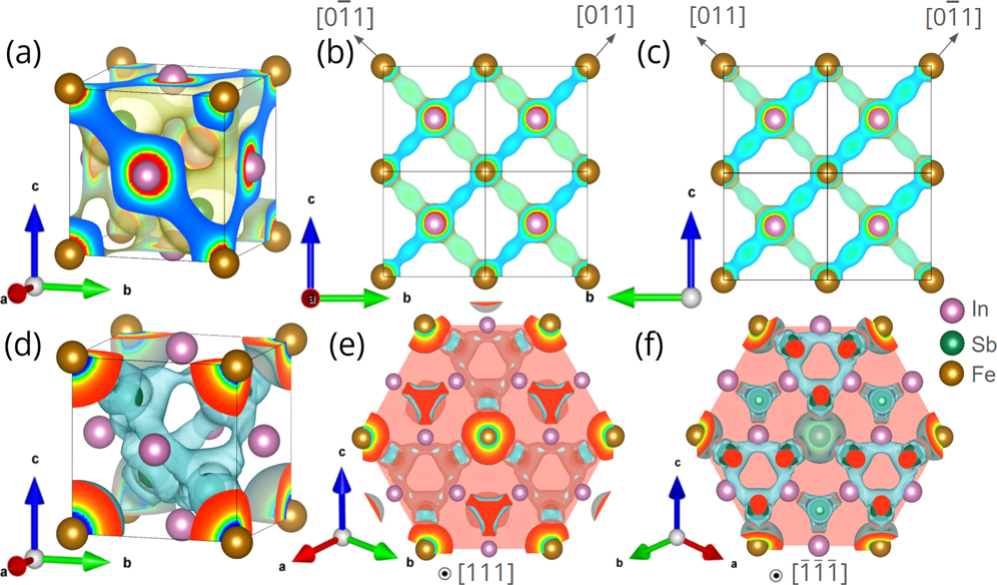
ECDs of In_0.75_Fe_0.25_Sb corresponding to (a) isosurface with 0.01500 e/bohr^3^, (b, c) representative isosurfaces with 0.03500 e/bohr^3^ in two-dimensional planar sections parallel to the (100) and (1̅00) crystallographic planes, respectively. In these planes, the hybrid orbitals exhibit the same lack of inversion symmetry of the zinc-blende structure. MMDs of In_0.75_Fe_0.25_Sb corresponding to (d) isosurface with 0.00055 e/bohr^3^, (e, f) representative isosurfaces with 0.00055 e/bohr^3^ in two-dimensional planar sections parallel to the (111) and (1̅1̅1̅) crystallographic planes, respectively. A percolative symmetric configuration of the magnetic moment is visible only on top of the (111) and (1̅1̅1̅) planes, which implies the existence of an easy magnetization axis in the crystallographic [111] direction. The choice of colors is arbitrary in each panel in order to facilitate visualization.

Representative partial MMD is shown in Figure 2d, whereas two-dimensional projective plots of the MMD structure are shown in Figure 2e,f. Due to the presence of BIA in In_0.75_Fe_0.25_Sb, the crystallographic directions [110] and [11̅0] cease to be symmetrically equivalent, exhibiting the same spacing but not the same atomic arrangements. Along these opposite directions, hybridizations occur between the 3d orbitals of Fe and 5p orbitals of Sb. This stabilizes ferromagnetism by forming spin-polarized interstitial charge bridges along these directions, rotated 90° relative to each other. The distribution of interstitial magnetic moments between the Fe atoms is symmetrical only along the crystallographic axis [111], which becomes the easy axis of magnetization. This simplified picture is corroborated by ab initio methods applied to determine the magnetic interactions between Fe atoms first, third and fourth nearest neighbors, which show that the shorter interactions are dominant in this system.^62^ Thus, the short contributions of ferromagnetic interactions spread and percolate through the whole crystal.

Most of the magnetic moment is centered on Fe atom sites, inducing a magnetocrystalline anisotropy due to the asymmetry along [110] and [11̅0]. This occurs when the magnetization rotate 90° from [111] toward these directions, as well as toward [001] and [100], as shown in Table 2. Thus an intrinsically anisotropic MMD is present in In_0.75_Fe_0.25_Sb, notably due to the Fe 3d - Sb 5p hybridization interstitial contribution. This is a quite interesting intrinsic magnetic property for development of applications in spintronics devices required for stabilizing magnetization orientation and subsequent switching control.^34,41^

**Table 2:** MAE Calculated for In_0.75_Fe_0.25_Sb^a^

crystallographic direction	MAE (kJ/m^3^)
[001]	3.33 × 10^2^
[100]	1.08 × 10^2^
[110]	5.84 × 10^2^
[11̅0]	1.04 × 10^3^
[111]	0

a All values are given relative to the value corresponding to the magnetization alignment parallel to the crystallographic [111] direction, which is the axis of easy magnetization.

The magnetocrystalline anisotropy energy (MAE) can be estimated by calculating the total energy *E* of the supercell when an external magnetic field is applied to orient the total magnetic moment.^32^ Thus, MAE was determined by applying the magnetic field along distinct orientations using the expression MAE = *E*_min_ – *E*_hkl_, where *E*_hkl_ is the energy corresponding to [hkl] directions and *E*_min_ is the energy corresponding to the axis of easy magnetization, namely along [111]. An important consequence of the incorporation of 25% Fe in the zinc-blende host, which naturally presents an inversion asymmetry, is that [111] remains as the only axis of symmetry.

For In_0.75_Fe_0.25_Sb, intermediate magnetization axes are [100] and [001], while hard axes are [110] and [11̅0]. The values of MAE obtained are not of the same order of magnitude as the experimental values of 3.9 kJ/m^3^ for 0.25 atom % Fe.^26^ This is mainly because the results presented are comparable with ideal and epitaxial samples. The real samples described in the literature^26,51^ are not homogeneous, with a discussion being made about the superparamagnetic behavior of Fe-rich domains present in the samples.^26^ Therefore, the extrinsic effects of compressive and tensile stresses represent negligible contributions to the magnetocrystalline anisotropy intrinsically induced by BIA.

Another important feature of these FMS is the presence of empty states above *E*_F_, which can be accessed by electrons from occupied states with opposite spin via absorption processes, generating a MCD signal. Intraband excitations involving transitions between Fe 3d orbitals with opposite spin states are predominant under IR and visible light excitation and the high density of majority spin states immediately above *E*_F_ favors the appearance of significant magneto-optical effects.

In the DFT theoretical framework used here, the complex optical conductivity σ = σ_R_ + iσ_I_ with its real (Re σ = σ_R_) and imaginary (Im σ = σ_I_) parts allows us to understand the relationship between the induced current density in the material and the magnitude of excitation electric fields at arbitrary frequencies.^63^ In other words, it generalizes electrical conductivity to the static limit of time independent or slowly varying electric fields.

In the configuration in which the magnetization is aligned with the crystallographic *z* axis (normal to the surface), the MCD can be expressed as the ratio of the imaginary part of off-diagonal σ_*xy*_ and real part of diagonal σ_*xx*_ components of the optical conductivity tensor (in rad):

MCD=Imσxy(ω)Reσxx(ω) 1

Our calculated results for MCD for photon energies from 1 to 4 eV are shown in Figure 3a. Significant peaks of MCD are observed for this excitation energy range, which are in very reasonable agreement with the experimental data from the MCD spectra of samples with *x* = 0.20 and 0.30 ^26^ considering a rigid shift of 0.8 eV. Experimentally, possible explanations to energy shifts in MCD spectra are quasi-static strain induced by laser pulses and band filling effects induced by laser pumping. This may reduce d-band filling, allowing a greater energetic range above the Fermi energy into which electrons can be excited. Discrepancies between theoretical calculations and empirical MCD spectra can be attributed to the difficulty in handling many-body corrections and vibronic coupling effects when nuclear motion influences electronic transitions.It is still unclear whether this rigid shift is systematic or dependent on the material’s composition, with further studies being required to clarify this point. Despite this, similar spectral features are also observed for samples with *x* = 0.16 ^33^ and *x* = 0.13 ^25^ in the same energy range.

**Figure 3:**
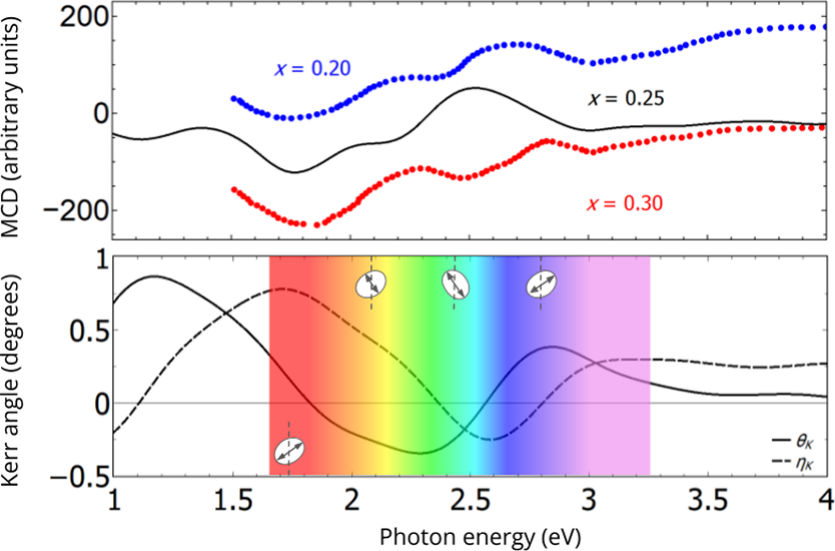
Spectroscopic plots of (a) MCD, (b) θ_K_ and η_K_ obtained using the same standard thermalization parameters in DFT calculations. A good agreement of the calculated MCD data with experimental results^26^ is obtained considering a shift of 0.8 eV. The sense of Kerr rotation and Kerr ellipticity with respect to the direction of magnetization for positive (clockwise) and negative (counterclockwise) values of θ_K_ and η_K_. Experimental data reprinted with permission from Tu, N. T.; Hai, P. N.; Anh, L. D.; Tanaka, M. Appl. Phys. Express 2019, 12 (10), 103004 (10.7567/1882-0786/ab3f4b). The Japan Society of Applied Physics. Reproduced by permission of IOP Publishing Ltd. All rights reserved.

The specific magnitudes of MCD are due to the differential absorption of left and right circularly polarized light which is induced in a sample in the presence of a magnetic field. Then, MCD spectral components for absorption of photons with energy between 1.2 and 3.2 eV are mainly due to intraband transitions between Fe 3d minority spin and majority spin interstitial p-d states that can be easily accessed immediately above *E*_F_ in the conduction band. The similarity of the MCD data for samples with *x* values between 0.20 and 0.30, which exhibit ferromagnetism above room temperature, to a certain degree translates the robustness of the magnetism associated with the asymmetry of occupation and localization of the Fe 3d orbitals in the DOS.

When a beam of plane-polarized light traveling in the *z* direction is incident parallel to the magnetization direction, the result is the appearance of a reflected beam with an optical Kerr rotation angle θ_K_ and a corresponding Kerr ellipticity η_K_. This in turn can be resolved into two circularly polarized beams with the spinning direction of the electric field parallel (clockwise) and antiparallel (counterclockwise) to magnetization,^64^ corresponding to the positive and negative signs of the Kerr rotations, respectively. The resulting complex Kerr angle Φ_K_ is^65^

$$\Phi_{K}=\theta_{K}+i\eta_{K}=-\frac{\sigma_{xy}/\sigma_{xx}}{\sqrt{1+i\sigma_{xx}/\omega }}$$ 2

Complex Kerr angle components for photon energies from 1 to 4 eV are shown in Figure 3b. Significant signal changes of Kerr rotation θ_K_ and ellipticity η_K_ are observed from IR to ultraviolet (UV) range through visible radiation. A continuous control of the sign of θ_K_, from positive to negative around 1.8 eV (from near-IR to visible) and negative to positive around 2.6 eV (from green to blue), remaining with this same signal until the UV region. Concomitantly, ellipticity follows the same trend as θ_K_, but with sign changes at different energy values.

A conventional Kerr gate device arranged between two crossed polarizers blocks all passing light except when a “gate” laser pulse passes through the Kerr medium, inducing a temporary anisotropic birefringence that rotates the polarization of the signal light.^65^ In other words, it allows some of the light to pass through the second perpendicular polarizer for a short time determined by the duration of the gate pulse. Kerr gate devices are primarily compared based on the Kerr medium material and the gating technique employed, which significantly affect performance characteristics like temporal resolution, signal efficiency and spectral range. For a conventional optical Kerr gate based on the use of a birefringent crystal for ultrashort switching times, the performance is limited by an inherent trade-off between high-signal transmittance and fast switching time even with a slow-response optical Kerr medium, which limits applications.

The Kerr device proposed here and schematized in Figure 4 relies on the Kerr dispersion property and the presence of magnetic Kerr rotation angle inversions in the IR to UV range. At extinction, the higher throughput expected is limited by the set of polarizers with Δθ_K_ varying from +0.3° to −0.3° in usable spectral range from 1.7 to 2.0 and 2.3 to 2.7 eV for near-infrared and violet radiations, respectively. The saturation magnetic field required for inversion of Kerr rotation signs is less than 1.3 T, which is the estimated anisotropy field. The thickness of the In_0.75_Fe_0.25_Sb film on the device needs to be greater than the optical penetration depth, estimated between 20 and 30 nm according to the DFT calculations. Using red or blue light such that θ_K_ = 0, corresponding approximately to 1.8 or 2.6 eV, the signal will be blocked when passing through the two crossed polarizers. However, when a visible-light pumping laser is incident on the ferromagnetic semiconductor, electrons from lower-energy states are excited to 3d majority spin states. The deexcitation processes generate the emission of reflected light with Kerr polarization, allowing some of it to pass through the second polarizer during the brief interval of the gate pulse.

**Figure 4:**
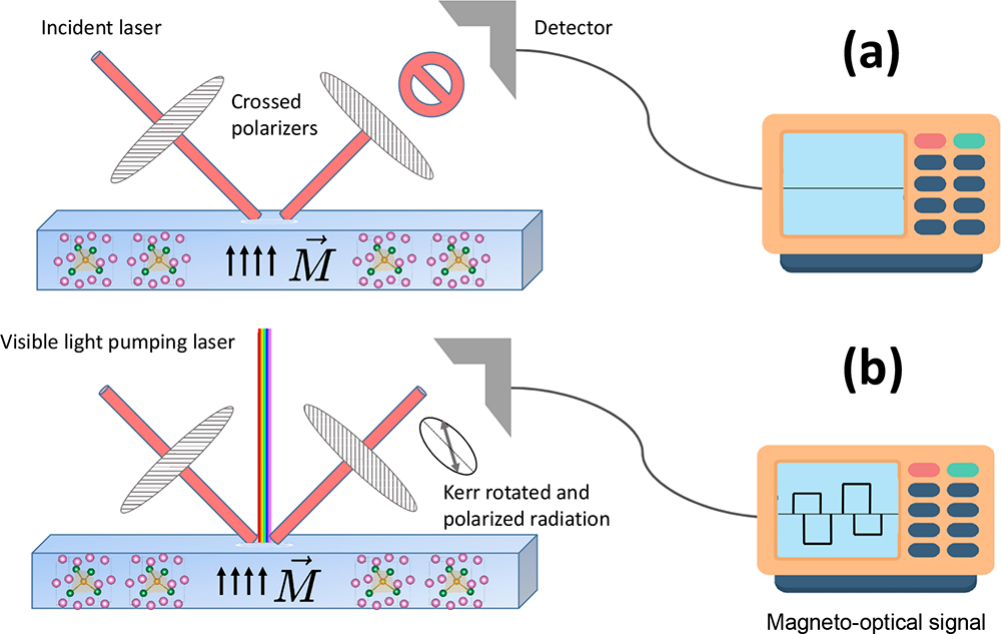
Simple schematic illustrating the proposed polar magneto-optical Kerr gate operation. (a) The crossed polarizers block incoming laser radiation with the energy set such that θ_K_ = 0 and thus no signal is detected. (b) When a visible light pumping laser is incident on the material, reflected Kerr rotated and polarized radiation is able to pass through the second polarizer only for the duration of the pulse, effectively constituting a gate.

Effectively, the “gate” is opened with a signal measured at that precise moment. The magneto-optical Kerr effect is induced by a short gate pulse triggered in the ferromagnetic semiconductor, generating a polarized light signal whose Kerr rotation angle can be reversed if the ferromagnetic semiconductor has its remnant magnetization reversed. This gating process has time-reversal symmetry at femtosecond range. Therefore, In_0.75_Fe_0.25_Sb is a potential candidate for applications in all-optical polarization controllers for photonic devices that require fast response times beyond thermally actuated polarization controllers.^66^ For example, a two-mode controlled phase rotation Kerr gate with applications in quantum computing at terahertz frequencies.^67^

## 3 Conclusions

In summary, the presented results elucidate the main magnetic and magneto-optical properties of the ferromagnetic semiconductor InFeSb homogeneously doped with isoelectronic iron impurities, exhibiting ferromagnetism even above room temperature. The magnetic moments are mainly centered on Fe atom sites which together with Sb 5p and Fe 3d hybridizations imposed by BIA of the zinc-blende structure results in an intrinsic magnetocrystalline anisotropy. As a consequence, the easy axis of magnetization is along the [111] crystallographic axis, the only one inversion symmetry axis preserved. Calculated MCD are in good agreement with trends observed in experimental results for samples In_0.80_Fe_0.20_Sb and In_0.70_Fe_0.30_Sb. Spectroscopic plots of calculated Kerr rotation angle and Kerr ellipticity reveal signal oscillatory behaviors from IR to UV range. These properties are suitable for applications in spintronic devices based on the spin-transfer torque effect as well as all-optical control of light polarization which is a requirement for all-optical tunable polarization rotation devices.

We hope that this work will attract attention for further studies on this interesting ferromagnetic semiconductor material, as well as other semiconductors with zinc-blende structure highly doped with ferromagnetic transition metals.

## References

[1] Ohno H. (1998). Making Nonmagnetic
Semiconductors Ferromagnetic. Science.

[2] Ohno H. (1999). Properties
of Ferromagnetic III–V Semiconductors. J. Magn. Magn. Mater..

[3] Sato K., Bergqvist L., Kudrnovský J., Dederichs P. H., Eriksson O., Turek I., Sanyal B., Bouzerar G., Katayama-Yoshida H., Dinh V. A., Fukushima T., Kizaki H., Zeller R. (2010). First-Principles Theory of Dilute
Magnetic Semiconductors. Rev. Mod. Phys..

[4] Tanaka M., Ohya S., Nam Hai P. (2014). Recent Progress
in III-V Based Ferromagnetic
Semiconductors: Band Structure, Fermi Level, and Tunneling Transport. Applied Physics Reviews.

[5] Dietl T., Ohno H. (2014). Dilute Ferromagnetic Semiconductors: Physics and Spintronic Structures. Rev. Mod. Phys..

[6] Dietl T., Ohno H., Matsukura F., Cibert J., Ferrand D. (2000). Zener Model
Description of Ferromagnetism in Zinc-Blende Magnetic Semiconductors. Science.

[7] Ohno Y., Young D. K., Beschoten B., Matsukura F., Ohno H., Awschalom D. D. (1999). Electrical
Spin Injection in a Ferromagnetic
Semiconductor Heterostructure. Nature.

[8] Fiederling R., Keim M., Reuscher G., Ossau W., Schmidt G., Waag A., Molenkamp L. W. (1999). Injection
and Detection of a Spin-Polarized
Current in a Light-Emitting Diode. Nature.

[9] Flatté M. E., Vignale G. (2001). Unipolar Spin Diodes
and Transistors. Appl. Phys. Lett..

[10] Flatté M. E., Yu Z. G., Johnston-Halperin E., Awschalom D. D. (2003). Theory
of Semiconductor Magnetic Bipolar Transistors. Appl. Phys. Lett..

[11] Chiba D., Yamanouchi M., Matsukura F., Ohno H. (2003). Electrical Manipulation
of Magnetization Reversal in a Ferromagnetic Semiconductor. Science.

[12] Yamanouchi M., Chiba D., Matsukura F., Ohno H. (2004). Current-Induced Domain-Wall
Switching in a Ferromagnetic Semiconductor Structure. Nature.

[13] Xiao M., Martin I., Yablonovitch E., Jiang H. W. (2004). Electrical Detection
of the Spin Resonance of a Single Electron in a Silicon Field-Effect
Transistor. Nature.

[14] Datta S. (2005). Proposal for
a “Spin Capacitor”. Appl. Phys.
Lett..

[15] Zunger A., Lany S., Raebiger H. (2010). The Quest for Dilute Ferromagnetism
in Semiconductors: Guides and Misguides by Theory. Physics.

[16] Coey J. M. D., Stamenov P., Gunning R. D., Venkatesan M., Paul K. (2010). Ferromagnetism in Defect-Ridden Oxides
and Related Materials. New J. Phys..

[17] Chen L., Yang X., Yang F., Zhao J., Misuraca J., Xiong P., von Molnár S. (2011). Enhancing
the Curie Temperature of
Ferromagnetic Semiconductor (Ga,Mn)As to 200 K via Nanostructure Engineering. Nano Lett..

[18] Schmidt G., Ferrand D., Molenkamp L. W., Filip A. T., van Wees B. J. (2000). Fundamental
Obstacle for Electrical Spin Injection from a Ferromagnetic Metal
into a Diffusive Semiconductor. Phys. Rev. B.

[19] Fert A., Jaffrès H. (2001). Conditions
for Efficient Spin Injection from a Ferromagnetic
Metal into a Semiconductor. Phys. Rev. B.

[20] Tanaka M., Higo Y. (2001). Large Tunneling Magnetoresistance
in GaMnAs/AlAs/GaMnAs Ferromagnetic
Semiconductor Tunnel Junctions. Phys. Rev. Lett..

[21] Tu N. T., Hai P. N., Anh L. D., Tanaka M. (2015). Magnetic Properties
and Intrinsic Ferromagnetism in (Ga,Fe)Sb Ferromagnetic Semiconductors. Phys. Rev. B.

[22] Tu N. T., Hai P. N., Anh L. D., Tanaka M. (2016). High-Temperature
Ferromagnetism
in Heavily Fe-doped Ferromagnetic Semiconductor (Ga,Fe)­Sb. Appl. Phys. Lett..

[23] Kudrin A. V., Lesnikov V. P., Pavlov D. A., Usov Yu. V., Danilov Yu. A., Dorokhin M. V., Vikhrova O. V., Milin V. E., Kriukov R. N., Kuznetsov Yu. M., Trushin V. N., Sobolev N. A. (2019). Formation of Epitaxial *p*-*i*-*n* Structures on the
Basis of (In,Fe)Sb and (Ga,Fe)Sb Diluted Magnetic Semiconductors Layers. J. Magn. Magn. Mater..

[24] Tu N. T., Hai P. N., Anh L. D., Tanaka M. (2018). Electrical
Control
of Ferromagnetism in the N-Type Ferromagnetic Semiconductor (In,Fe)­Sb
with High Curie Temperature. Appl. Phys. Lett..

[25] Kudrin A. V., Danilov Y. A., Lesnikov V. P., Dorokhin M. V., Vikhrova O. V., Pavlov D. A., Usov Y. V., Antonov I. N., Kriukov R. N., Alaferdov A. V., Sobolev N. A. (2017). High-Temperature Intrinsic Ferromagnetism
in the (In,Fe)Sb Semiconductor. J. Appl. Phys..

[26] Tu N. T., Hai P. N., Anh L. D., Tanaka M. (2019). Heavily Fe-doped Ferromagnetic
Semiconductor (In,Fe)Sb with High Curie Temperature and Large Magnetic
Anisotropy. Applied Physics Express.

[27] Pillai A., Goel S., Anh L. D., Tanaka M. (2023). Control of Magnetic
Anisotropy by Epitaxial Strain in the n-Type Ferromagnetic Semiconductor
(In,Fe)­Sb. Phys. Rev. B.

[28] Nam
Hai P., Duc Anh L., Mohan S., Tamegai T., Kodzuka M., Ohkubo T., Hono K., Tanaka M. (2012). Growth and Characterization
of N-Type Electron-Induced Ferromagnetic Semiconductor (In,Fe)­As. Appl. Phys. Lett..

[29] Kudrin A. V., Danilov Yu. A., Lesnikov V. P., Pitirimova E. A. (2016). Nonlinear
Room-Temperature Hall Effect in n-InFeAs Layers. Technical Physics Letters.

[30] Anh L. D., Hai P. N., Tanaka M. (2016). Observation of Spontaneous
Spin-Splitting
in the Band Structure of an n-Type Zinc-Blende Ferromagnetic Semiconductor. Nat. Commun..

[31] Kudrin A. V., Lesnikov V. P., Danilov Y. A., Dorokhin M. V., Vikhrova O. V., Demina P. B., Pavlov D. A., Usov Y. V., Milin V. E., Kuznetsov Y. M., Kriukov R. N., Konakov A. A., Tabachkova N. Y. (2020). High-Temperature
Intrinsic Ferromagnetism in Heavily Fe-doped GaAs Layers. Semicond. Sci. Technol..

[32] Zarpellon J., Mosca D. H., Varalda J. (2024). Ferromagnetism in Heavily Fe-doped
GaAs: A DFT Study. Phys. Scr..

[33] Tu N. T., Hai P. N., Anh L. D., Tanaka M. (2018). High-Temperature Ferromagnetism
in New n-Type Fe-doped Ferromagnetic Semiconductor (In,Fe)­Sb. Applied Physics Express.

[34] Okano R., Hotta T., Takeda T., Araki K., Takase K., Anh L. D., Sakamoto S., Takeda Y., Fujimori A., Tanaka M., Kobayashi M. (2023). Ferromagnetism Induced by Hybridization
of Fe 3d Orbitals with Ligand InSb Bands in the n-Type Ferromagnetic
Semiconductor (In,Fe)­Sb. Phys. Rev. B.

[35] Goel S., Anh L. D., Ohya S., Tanaka M. (2019). Ferromagnetic Resonance
and Control of Magnetic Anisotropy by Epitaxial Strain in the Ferromagnetic
Semiconductor (Ga_0.8_Fe_0.2_) at Room Temperature. Phys. Rev. B.

[36] Kudrin A. V., Lesnikov V. P., Kriukov R. N., Danilov Y. A., Dorokhin M. V., Yakovleva A. A., Tabachkova N. Y., Sobolev N. A. (2023). Multilayer Epitaxial
Heterostructures with Multi-Component III–V:Fe Magnetic Semiconductors. Nanomaterials.

[37] Thanh T. N., Otsuka T., Arakawa Y., Anh L. D., Tanaka M., Hai P. N. (2022). Spin Transport in Fully Ferromagnetic p–n Junctions. J. Appl. Phys..

[38] Anh L. D., Hai P. N., Tanaka M. (2018). Electrical
Tuning of the Band Alignment
and Magnetoconductance in an n-Type Ferromagnetic Semiconductor (In,Fe)­As-based
Spin-Esaki Diode. Appl. Phys. Lett..

[39] Hu L., Gao J., Shen S.-Q. (2004). Conductance Modulations in Spin Field-Effect Transistors
under Finite Bias Voltages. Phys. Rev. B.

[40] Jiang K.-M., Zhang R., Yang J., Yue C.-X., Sun Z.-Y. (2010). Tunneling
Magnetoresistance Properties in Ballistic Spin Field-Effect Transistors. IEEE Trans. Electron Devices.

[41] Jungwirth T., Niu Q., MacDonald A. H. (2002). Anomalous
Hall Effect in Ferromagnetic Semiconductors. Phys. Rev. Lett..

[42] Lang R., Winter A., Pascher H., Krenn H., Liu X., Furdyna J. K. (2005). Polar Kerr Effect Studies of Ga_1‑x_Mn_x_As Epitaxial Films. Phys. Rev.
B.

[43] Rozkotová E., Němec P., Tesařová N., Malý P., Novák V., Olejník K., Cukr M., Jungwirth T. (2008). Coherent Control
of Magnetization Precession in Ferromagnetic Semiconductor (Ga,Mn)­As. Appl. Phys. Lett..

[44] Mitsumori Y., Oiwa A., Słupinski T., Maruki H., Kashimura Y., Minami F., Munekata H. (2004). Dynamics of
Photoinduced Magnetization
Rotation in Ferromagnetic Semiconductor p-(Ga,Mn)­As. Phys. Rev. B.

[45] Yao H., Yusuf Günel H., Blömers C., Weis K., Chi J., Grace L. J., Liu J., Grützmacher D., SchäPers T. (2012). Phase Coherent Transport in InSb
Nanowires. Appl. Phys. Lett..

[46] Fan D., Kang N., Ghalamestani S. G., Dick K. A., Xu H. Q. (2016). Schottky
Barrier and Contact Resistance of InSb Nanowire Field-Effect Transistors. Nanotechnology.

[47] Nilsson H. A., Karlström O., Larsson M., Caroff P., Pedersen J. N., Samuelson L., Wacker A., Wernersson L.-E., Xu H. Q. (2010). Correlation-Induced
Conductance Suppression at Level Degeneracy in
a Quantum Dot. Phys. Rev. Lett..

[48] Fan D., Li S., Kang N., Caroff P., Wang L. B., Huang Y. Q., Deng M. T., Yu C. L., Xu H. Q. (2015). Formation of Long
Single Quantum Dots in High Quality InSb Nanowires Grown by Molecular
Beam Epitaxy. Nanoscale.

[49] Nadj-Perge S., Pribiag V. S., van den
Berg J. W. G., Zuo K., Plissard S. R., Bakkers E. P. A. M., Frolov S. M., Kouwenhoven L. P. (2012). Spectroscopy
of Spin-Orbit Quantum Bits in Indium Antimonide Nanowires. Phys. Rev. Lett..

[50] Pribiag V. S., Nadj-Perge S., Frolov S. M., van den Berg J. W. G., van Weperen I., Plissard S. R., Bakkers E. P. a. M., Kouwenhoven L. P. (2013). Electrical
Control of Single Hole Spins in Nanowire
Quantum Dots. Nat. Nanotechnol..

[51] Kudrin A. V. (2019). Robustness of Ferromagnetism
in (In,Fe)Sb Diluted Magnetic Semiconductor
to Variation of Charge Carrier Concentration. J. Magn. Magn. Mater..

[52] See http://elk.sourceforge.net/ for “The Elk Code”.

[53] Perdew J. P., Ruzsinszky A., Csonka G. I., Vydrov O. A., Scuseria G. E., Constantin L. A., Zhou X., Burke K. (2008). Restoring the Density-Gradient
Expansion for Exchange in Solids and Surfaces. Phys. Rev. Lett..

[54] Blöchl P. E., Jepsen O., Andersen O. K. (1994). Improved
Tetrahedron Method for Brillouin-zone
Integrations. Phys. Rev. B.

[55] Vinet P., Rose J. H., Ferrante J., Smith J. R. (1989). Universal Features
of the Equation of State of Solids. J. Phys.:
Condens. Matter.

[56] Vinogradova K. I., Kolchanova N. M., Mironov I. F., Nasledov D. N., Smetannikova Yu. S., Yarmarkin V. K. (1972). On the Distribution of Fe and Co in InSb and GaSb at
Zone Melting. physica status solidi (a).

[57] Zhang P., Kim Y.-H., Wei S.-H. (2019). Origin
of High-TC Ferromagnetism
in Isovalent-Doped III-V Semiconductors. Physical
Review Applied.

[58] Dietl T., Ohno H., Matsukura F. (2001). Hole-Mediated
Ferromagnetism in Tetrahedrally
Coordinated Semiconductors. Phys. Rev. B.

[59] Berciu M., Bhatt R. N. (2001). Effects of Disorder
on Ferromagnetism in Diluted Magnetic
Semiconductors. Phys. Rev. Lett..

[60] Ohya S., Muneta I., Xin Y., Takata K., Tanaka M. (2012). Valence-Band
Structure of Ferromagnetic Semiconductor (In,Ga,Mn)­As. Phys. Rev. B.

[61] You J.-Y., Gu B., Maekawa S., Su G. (2020). Microscopic
Mechanism of High-Temperature
Ferromagnetism in Fe, Mn, and Cr-doped InSb, InAs, and GaSb Magnetic
Semiconductors. Phys. Rev. B.

[62] Shinya H., Fukushima T., Masago A., Sato K., Katayama-Yoshida H. (2018). First-Principles
Prediction of the Control of Magnetic Properties in Fe-doped GaSb
and InSb. J. Appl. Phys..

[63] Sinova J., Jungwirth T., Kučera J., MacDonald A. H. (2003). Infrared
Magneto-Optical Properties of (III,Mn)V Ferromagetic Semiconductors. Phys. Rev. B.

[64] Araujo R. M. T., Zarpellon J., Mosca D. H., Varalda J. (2025). Magneto-Optical
Properties
of Quasi-Two-Dimensional MnGaGe and MnAlGe Ferromagnets. J. Alloys Compd..

[65] Ravindran P., Delin A., James P., Johansson B., Wills J. M., Ahuja R., Eriksson O. (1999). Magnetic, Optical,
and Magneto-Optical Properties of MnX (X = As, Sb, or Bi) from Full-Potential
Calculations. Phys. Rev. B.

[66] Moroney N., Del Bino L., Zhang S., Woodley M. T. M., Hill L., Wildi T., Wittwer V. J., Südmeyer T., Oppo G.-L., Vanner M. R., Brasch V., Herr T., Del’Haye P. (2022). A Kerr Polarization Controller. Nat. Commun..

[67] Budinger N., Furusawa A., van Loock P. (2024). All-Optical
Quantum Computing Using
Cubic Phase Gates. Physical Review Research.

